# Association of Polyaminergic Loci With Anxiety, Mood Disorders, and Attempted Suicide

**DOI:** 10.1371/journal.pone.0015146

**Published:** 2010-11-30

**Authors:** Laura M. Fiori, Brigitte Wanner, Valérie Jomphe, Jordie Croteau, Frank Vitaro, Richard E. Tremblay, Alexandre Bureau, Gustavo Turecki

**Affiliations:** 1 McGill Group for Suicide Studies, Douglas Mental Health University Institute, McGill University, Montreal, Quebec, Canada; 2 Research Unit on Children's Psychosocial Maladjustment, Université de Montréal, Montreal, Quebec, Canada; 3 Centre de recherche Université Laval Robert-Giffard, Université Laval, Quebec City, Quebec, Canada; 4 School of Public Health and Population Sciences, University College Dublin, Dublin, Ireland; University of Muenster, Germany

## Abstract

**Background:**

The polyamine system has been implicated in a number of psychiatric conditions, which display both alterations in polyamine levels and altered expression of genes related to polyamine metabolism. Studies have identified associations between genetic variants in spermidine/spermine N1-acetyltransferase (SAT1) and both anxiety and suicide, and several polymorphisms appear to play important roles in determining gene expression.

**Methodology/Principal Findings:**

We genotyped 63 polymorphisms, spread across four polyaminergic genes (SAT1, spermine synthase (SMS), spermine oxidase (SMOX), and ornithine aminotransferase like-1 (OATL1)), in 1255 French-Canadian individuals who have been followed longitudinally for 22 years. We assessed univariate associations with anxiety, mood disorders, and attempted suicide, as assessed during early adulthood. We also investigated the involvement of gene-environment interactions in terms of childhood abuse, and assessed internalizing and externalizing symptoms as endophenotypes mediating these interactions. Overall, each gene was associated with at least one main outcome: anxiety (SAT1, SMS), mood disorders (SAT1, SMOX), and suicide attempts (SAT1, OATL1). Several SAT1 polymorphisms displayed disease-specific risk alleles, and polymorphisms in this gene were involved in gene-gene interactions with SMS to confer risk for anxiety disorders, as well as gene-environment interactions between childhood physical abuse and mood disorders. Externalizing behaviors demonstrated significant mediation with regards to the association between OATL1 and attempted suicide, however there was no evidence that externalizing or internalizing behaviors were appropriate endophenotypes to explain the associations with mood or anxiety disorders. Finally, childhood sexual abuse did not demonstrate mediating influences on any of our outcomes.

**Conclusions/Significance:**

These results demonstrate that genetic variants in polyaminergic genes are associated with psychiatric conditions, each of which involves a set of separate and distinct risk alleles. As several of these polymorphisms are associated with gene expression, these findings may provide mechanisms to explain the alterations in polyamine metabolism which have been observed in psychiatric disorders.

## Introduction

Mood and anxiety disorders represent the two most common forms of mental illness, and are associated with a wide range of behavioural and somatic symptoms as well as substantial disability and decreased quality of life [1]. Suicidal behaviors are closely, but not exclusively, associated with both psychiatric disorders [2]–[5]. These behaviors, which comprise ideation, attempts, and completed suicide, are amongst the most devastating consequences of psychiatric disorders, and account for over a million deaths worldwide each year [6]. Over the years, the importance of genetic factors in psychiatric disorders has become increasingly apparent, with overall heritability rates for depressive disorders, anxiety disorders, and suicide ranging between 30–50% [7]–[11], involving both shared and distinct genetic vulnerabilities [12]–[15]. Substantial efforts have been put towards identifying genes and pathways involved in the pathology and etiology of these conditions as these both represent sites involved in conferring risk for their development, as well as act as potential targets for pharmaceutical treatments [16], [17]. Although the substantial evidence emerging from genetic, metabolic, and pharmacological studies investigating these disorders has implicated the involvement of monoaminergic neurotransmission, particularly the serotonin and catecholamine systems, dysregulation of these systems is not sufficient to account for all aspects of the clinical presentations or heritability associated with these disorders, and it has become abundantly clear that additional systems are involved.

The polyamine system represents an important source for neurobiological factors involved in mood disorders, anxiety disorders, and suicide. In recent years, the majority of the focus has been on its involvement in suicidal behaviours, yet considerable research across the last three decades has pointed towards roles for the polyamine system in several psychiatric conditions, including schizophrenia, mood disorders, and anxiety disorders, and particular emphasis has been placed on the importance of this system in the physiological and behavioural responses to stress [18].

The polyamines are ubiquitous aliphatic molecules, comprising spermine, spermidine, putrescine, and agmatine, which are involved in a vast range of cellular functions, including cell cycle modulation, scavenging reactive oxygen species, control of gene expression, and possess important roles in neurotransmission through their modulation of the functioning of cell-surface receptors, involvement in intracellular signalling pathways, as well as their putative roles as neurotransmitters [19], [20]. Cellular levels of the polyamines are extensively regulated through tight control of their biosynthesis, catabolism, and transport, and much of the evidence for their involvement in psychiatric conditions to date has revolved around variations in the levels of the polyamines, as well as alterations in the expression of genes involved in polyamine metabolism, including spermidine/spermine N1-acetyltransferase (SAT1), spermine oxidase (SMOX), spermine synthase (SMS), and ornithine aminotransferase–like 1 (OATL1) [21]–[26]. Given the extensive molecular functions of the polyamines, their precise roles in the etiology and pathology of psychiatric disorders remain unclear, although evidence from animal studies have suggested that at least some of their antidepressant and anxiolytic effects involve modulation of transmission through N-methyl-D-aspartate receptors, α2-adrenoceptors, imidazoline receptors, and serotonin receptors [18].

In spite of the strong evidence suggesting a role for the polyamine system in depression, anxiety, and suicide, with recent evidence indicating direct polyamine dysregulation in brain tissue from individual who died by suicide [27], only a few studies have investigated polyamine-related genes at the genetic level, all of which have focused exclusively on promoter polymorphisms in SAT1, the main rate-limiting enzyme in polyamine catabolism. Three studies found significant associations: rs6526342 with suicide [21], rs6151267 with suicide in depressed individuals [28], and rs1960264 with anxiety [29]. However, another study found no association between either rs6526342 or rs17286006 and suicide [23], nor was rs1960264 found to be associated with schizophrenia [30]. Interestingly, these polymorphisms are part of a larger haplotype block which is associated with SAT1 expression in the brain [31], thereby representing a link between genetic variability and downstream functional consequences. To date, no genetic studies have examined the relationships between polymorphisms in other polyamine-related genes and psychiatric disorders.

The aim of the present study was to expand our understanding of the relationship between polymorphisms in polyamine-related genes and psychiatric disorders, in particular mood disorders, anxiety disorders, and suicidal behaviours, as well as to investigate the potential for epistasis between genetic risk factors, and to identify variables which may influence the effects of genetics on psychopathology. To this end, we genotyped a large number of polymorphisms in several genes involved in polyamine metabolism in a French-Canadian cohort which has been followed for over 20 years. During this time, substantial clinical and epidemiological measures were collected, and a number of these were assessed as potential mediators for our genetic associations. Given the influence of environmental stressors on the polyamine system [18], [32] as well as consistent findings implicating early life adversity in the development of psychiatric disorders [33]–[35], the influence of childhood sexual and physical abuse was assessed. We also investigated several personality measures, comprising externalizing or internalizing behaviors, as endophenotypes for our main outcomes. As our previous research investigating externalizing and internalizing trajectories in young children (ages 6 to 12) did not identify mediating effects with regards to mood disorders or suicide attempts [36], [37], in this study these symptoms were investigated at an older age (adolescence).

Overall, we identified several genetic risk factors associated with mood disorders, anxiety disorders, and suicidal behaviours, as well as several clinical variables which mediate these effects.

## Methods

### Study Participants

Participants in this study were part of a larger cohort of French-Canadians recruited in 1986–1987, then followed-up for over 20 years. A more detailed description of this cohort as well as the assessment schedules is found in [34], [36]. In brief, children were recruited from francophone schools in Quebec at age 6, where they were assessed through a variety of demographic, social and behavioral measures in several waves, representing childhood, mid-adolescence, early adulthood, and mid-adulthood. The initial sample comprised 3017 children, of which 2000 were randomly selected and are considered representative of the young French-speaking population. DNA was collected from the 1255 respondents among the initial sample. In the representative sample of 2000 subjects, there were no differences between the respondents and nonrespondents for parental age at birth of first child, maternal socioeconomic status, or proportion living with both biological parents. As both family adversity (described below) and gender were related to attrition in our previous studies in this cohort [36], we used them to construct weights for multivariate analyses. This study was approved by the institutional review boards of the University of Montreal and McGill University. Written informed consent was obtained from all subjects.

### Measures

#### Genetic Factors

Single nucleotide polymorphisms (SNPs) were selected from four polyamine-related genes: SAT1 (NM_002970), SMOX (NM_175839), SMS (NM_004595), and OATL1 (NM_001006113). SNPs were located between 5 kb upstream of the transcription start site to 5 kb downstream of the end of the last exon. Common tag SNPs (minor allele frequencies >5%) for each gene were selected using HapMap data for the Utah residents with Northern and Western European ancestry [38] and the multi-marker tagging procedure in Tagger (r^2^>0.8) [39]. Additional SNPs in the upstream regions were selected using the NCBI, Pupa, and Ensembl databases. In total, 63 polyamine-related SNPs were genotyped, as shown in Supplementary Table S1. We also included 42 anonymous markers spread in non-coding regions across the genome in order to detect population stratification. Genotyping was performed using a 768-SNP Illumina platform with a custom-designed GoldenGate panel. Following genotyping, several quality control steps were performed as described in [36]. Two SNPs (rs1535225 and rs2238958) had call rates of less than 90% and were removed.

#### Environmental Factors

Among the overall study group, we defined two subsamples through their exposure to physical (CPA) or sexual (CSA) abuse in childhood (under 18 years of age). Subjects in the CPA group self-reported severe or very severe physical abuse perpetrated by either parent, as assessed in the Conflict Tactics Scales [40], [41]. Childhood sexual abuse was defined as incidences of sexual violence experienced before the age of 18, and was assessed by self-report as described in [42].

The effects of family adversity were also assessed. As described in [43], we computed a family adversity index based upon maternal reports regarding: (1) family structure (two parent or single), (2) educational level of both parents (or the parent with whom the child was living), (3) occupational status of both parents (or occupation of the parent with whom the child was living) based on the Blishen's occupational prestige scale [44] and, (4) mother's and father's age at birth of the first child. Higher values correspond to higher family adversity levels at the time when the participants were approximately 6 years of age.

### Mediators

#### Diagnostic Interview Schedule for Children (DIS-C) [45]

Using the DIS-C, self-reports of hyperactivity-impulsivity (8 symptoms), oppositional defiant disorder (9 symptoms), conduct disorder (11 symptoms), generalized anxiety (18 symptoms), panic disorder (13 symptoms), major depression (9 symptoms), and dysthymia (6 symptoms) were assessed. A total externalizing-disruptiveness score was obtained by summing the symptom counts associated with hyperactivity-impulsivity, oppositional defiant disorder, and conduct disorder (Cronbach alpha  = 0.77). Two separate internalizing scores were generated: an internalizing-anxiety score was calculated by summing generalized anxiety and panic disorder symptoms (Cronbach alpha  = 0.93), while an internalizing-depression score was generated by summing major depression and dysthymia symptoms (Cronbach alpha  = 0.94). To equally weight each disorder when calculating the total sum scores, each count variable was transformed to range between 0 and 1 (by dividing the total count by the maximum count after adding 1 as a constant) before calculating the total sum score.

### Covariates and Outcomes

#### Diagnostic Interview Schedule for Adults (DIS) [46]

This schedule assesses mood (major depression, bipolar disorder and dysthymia), anxiety (generalized anxiety, panic and phobias), disruptive (i.e., antisocial personality), and substance abuse disorders (abuse and/or dependence on drugs, alcohol and nicotine) using DSM-III-R criteria. Mood and anxiety disorders represented outcomes and, in addition, served together with disruptive and substance abuse disorders and suicide attempts (see below) as the covariate “history of psychopathology”, following previous research [36]. This count variable summarized the number of diagnoses in each individual. For each of the main outcome variables, the other two outcomes were part of this count variable, in addition to substance and disruptive disorders. By means of controlling for the other disorders, any significant gene-outcome relationship is independent of these potentially confounding effects. The DIS was also used to provide information regarding parental history of suicide attempts, anxiety, and mood disorders. If either parent had a positive history for these control variables, the respective history was coded as ‘1’, otherwise it was coded as ‘0’.

#### Suicide attempts

Suicide attempt status was based on both adolescent and adult assessments. Adolescent history was obtained from parental/adolescent responses to a question from the DIS-C [45]: ‘Have you already attempted suicide?’ Either parental or self-report was sufficient for a person to be classified as an attempter. Adult suicide attempts were assessed with a question from the Suicidal Intent Scale [47]: ‘Have you already attempted suicide?’ A positive attempt status was coded as ‘1’ and negative as ‘0’.

### Statistical Approach

#### Population Stratification

Although the French Canadians descended from a small number of individuals and displays a well-known founder effect [48], [49], we nonetheless felt it was necessary to identify population outliers - individuals displaying significantly different allele frequency distributions from the rest of the sample [50]. We used the genotype log likelihood test statistic with a cut-off of P = 0.01, identifying 12 outliers, which were excluded from subsequent analyses. All SNPs fulfilled Hardy-Weinberg equilibrium.

#### Univariate analyses

We first investigated direct effects exerted by SNPs and haplotypes on our main outcomes (suicide attempts, mood disorders, and anxiety disorders). We also postulated that CSA or CPA may create associations of genetic variants with phenotypes only in subjects exposed to these environments and, therefore screened for such moderating or interaction effects by testing for associations of SNPs with the phenotypes in the subsamples of subjects exposed to CSA or CPA.

Firstly, in order to identify redundant SNPs, we used the squared correlations between each SNP to identify all groups of SNPs with r^2^>0.99, then selected only one SNP from within each group of these perfectly correlated SNPs, yielding a set of 43 SNPs which were retained for all further analyses. The patterns of linkage disequilibrium between SNPs within each gene are shown in Supplementary Figure S1, and the set of non-redundant SNPs are displayed in Supplementary Table S1.

χ^2^-tests and Fisher's exact tests, in conjunction with a false detection rate (FDR) cutoff of ≤0.20, were used to identify significant SNPs under allelic, recessive and dominant genetic models with respect to the minor allele. For X-linked SNPs, males were coded as homozygous when assessing recessive and dominant models. The FDR attached to each P-value for suicide attempts in the total sample as well as all analyses in the CSA and CPA subsamples was estimated using the Efron [51] method implemented in R. The Benjamini-Hochsberg [52] method was used to compute FDRs for analyses of anxiety and depression in the total sample. The Benjamini-Hochsberg method was employed for these analyses as the empirical null distribution was heavily skewed and did not fit a normal distribution, which is required to apply the Efron method. SNPs with FDR corrected P-value ≤0.20 were further tested in adjusted multivariate models.

We determined haplotype blocks within each gene in the total sample using *entropy.blocker* [53] implemented in the R statistical environment. Global association tests to haplotypes within each haplotype block were performed for suicide, depression, and anxiety. For SMOX, haplotype analyses were performed using the function *haplo.score* [54] from the package *haplo.stats*, implemented in R. Analyses for the X chromosome genes were performed using UNPHASED [55]. The analysis of SMS haplotypes was more complicated due to the large number of SNPs in the SMS haplotype block. Firstly, we formed ten subgroups of strongly correlated SNPs, then made three different selections of ten SNPs by randomly picking one SNP in each subgroup. These three selections were then analysed using UNPHASED.

#### Multivariate analyses

Using a series of regression-based analyses adjusted for psychopathology, we retained all significant SNPs in a given gene (P<0.05), and included them together in a model across genes. We also tested if the results in the final models changed if we applied weights adjusting for the probability of remaining in the sample conditional on the variables related to attrition: gender and family adversity. The expectation-maximization method (EM) was used to impute missing covariate values.

We analyzed two forms of moderating effects: gene-gene interactions, and gene-environment interactions. Gene-gene interactive effects were assessed in order to examine moderation of the effects of polyaminergic loci on the three outcomes by other polyaminergic loci. These interactions were performed between pairs of SNPs, in separate genes, from among those that displayed significant univariate associations to the three main outcomes. To assess these effects, in each regression model, we included two of the significant main effects from the univariate analyses as well as their interaction term. Interactions were only assessed using SNPs which were significant in the univariate analyses, and only between SNPs of different genes. Each set of predictors was tested with a logistic and an additive regression model. We examined allelic–allelic, dominant–dominant, dominant–recessive, recessive–dominant and recessive–recessive model combinations, depending on the significant mode found on the univariate step. Specifically, we conducted ten regressions (suicide attempts: 2, mood disorders: 0, anxiety disorders: 8) and empirically determined whether logistic or additive link functions yielded a better fit to the data, resulting in two highly correlated sets of regressions and a total of twenty tests. We believe that the low statistical power to detect interaction effects in field studies, as described by McClelland and Judd [56], also applies to psychiatric and genetic studies. We therefore did not employ Type-I Error protection for the gene-gene interaction tests. Following similar procedures, we tested gene-environment interactions to examine the moderation of the effects of childhood abuse on the associations of SNPs with any of the three outcomes. We controlled for confounding effects of passive or evocative gene-environment correlations by adjusting models for parental histories of psychopathology, and by demonstrating that genotypes did not influence the exposure to abuse. Post-hoc tests were used to quantify regression slopes and examine the statistical significance of significant moderating effects [57]. Corrections for multiple testing were not performed at this stage in the analyses, as these tests were performed using only SNPs which passed our FDR criteria in the CSA and CPA subsamples prior to the inclusion of covariates. Power analyses for these tests with the mood disorder phenotype are shown in Supplementary Tables S2 and S3. As the prevalence of suicide attempts and anxiety disorders were higher than that of mood disorders in the sample, greater power is expected for these two phenotypes.

In the presence of covariates, count scores of externalizing and internalizing disorders were investigated as endophenotypes mediating the significant main effects identified in the final models of suicide attempts, mood disorders, and anxiety disorders. Specifically, mediation testing was performed to identify variables which accounted for some or all of the associations between our genotypes and main outcomes [58], and the significance of these results was assessed using Sobel and Goodman tests.

## Results

### Sample

From the initial 1255 subjects, fourteen subjects with call rates less than 95% were excluded, and 108 subjects were removed as they displayed non-White ethnicity. Assessment for population outliers identified 12 subjects displaying significantly different allele frequencies than the remainder of the population. After exclusions, the total analyzed sample consisted of 1121 (*N* = 664, 59% female) individuals, as shown in Table 1. Overall, exposure to CSA or CPA was significantly associated with higher rates of suicide attempts, mood disorders, and anxiety disorders.

**Table 1:** Characteristics of the total sample and the childhood sexual abuse (CSA) and childhood physical abuse (CPA) subsamples with respect to the main outcomes.

Sample		Suicide Attempts		Mood Disorders		Anxiety Disorders	
	N	N (%)	χ^2^ (1 df)	N (%)	χ^2^ (1 df)	N (%)	χ^2^ (1 df)
Total	1121	117 (10%)		107 (10%)		239 (21%)	
CSA	230	53 (23%)***	48.9	44 (19%)***	28.1	77 (33%)***	25.5
CPA	316	55 (17%)***	22.9	47 (15%)***	12.9	88 (28%)***	11.2

χ^2^ tests for the CSA and CPA subgroups were performed with respect to all other subjects not within those subgroups.

N =  number,

*** = P<0.001.

### Univariate analyses

Our first objective was to determine if genetic variants within these polyamine genes were associated with our main outcomes in order to identify potential risk or protective factors involved in suicide attempts, mood disorders, or anxiety disorders.

#### Individual SNPs

We first examined association of the 43 non-redundant SNPs with each of the three adult outcomes (mood disorders, anxiety disorders, and suicide attempts) under allelic, dominant, and recessive models. As shown in Table 2, two SAT1 and five SMS SNPs exhibited recessive modes of inheritance regarding the prediction of anxiety disorders, while three SMS SNPs were significant in the dominant model. These two SAT1 SNPs were also significantly associated with mood disorders, where they exhibited a dominant mode of inheritance. Additionally, six SNPs were found to be significantly associated with mood disorders in the CPA subsample in the dominant mode. Finally, three SNPs, including one in SAT1 and two in OATL1, were significantly associated with suicide attempts. Additionally, the results of the univariate analyses for the CSA subsample indicated no significant links to any of the adult outcomes, ruling out gene X environment interactions involving childhood sexual abuse.

**Table 2:** Significant univariate associations between polyamine genes and adult outcomes in the total and childhood physical abuse (CPA) samples.

Gene	SNP	FDR	Mode	Affected (%)	Unaffected (%)	Sample
**Anxiety Disorders**						
SAT1 **	rs6526342	0.14	recessive	0.221	0.234	Total
SAT1 **	rs3764885	0.14	recessive	0.217	0.220	Total
SMS *	rs5951672	0.19	dominant	0.071	0.050	Total
SMS *	rs2040357	0.19	recessive	0.333	0.344	Total
SMS *	rs732946	0.19	dominant	0.064	0.042	Total
SMS **	rs5904598	0.14	recessive	0.262	0.310	Total
SMS **	rs10521911	0.16	recessive	0.171	0.214	Total
SMS **	rs5951676	0.14	dominant	0.071	0.046	Total
SMS *	rs5951678	0.19	recessive	0.243	0.260	Total
SMS **	rs6654100	0.14	recessive	0.167	0.210	Total
**Mood Disorders**						
SAT1 **	rs6526342	0.19	dominant	0.283	0.224	Total
SAT1 **	rs3764885	0.19	dominant	0.262	0.214	Total
SAT1 ^A^ **	rs6526342	0.02	dominant	0.305	0.211	CPA
SAT1 ^A^ **	rs3764885	0.02	dominant	0.305	0.202	CPA
SAT1 *	rs1894289	0.04	dominant	0.451	0.366	CPA
SMOX^ A^ *	rs1622950	0.04	dominant	0.596	0.459	CPA
SMOX^ A^ *	rs1765017	0.04	dominant	0.564	0.447	CPA
SMOX^ A^ *	rs6084657	0.14	dominant	0.596	0.489	CPA
**Suicide Attempts**						
OATL1^A^ **	rs11795513	0.007	dominant	0.400	0.330	Total
OATL1 *	rs2249583	0.03	dominant	0.460	0.411	Total
SAT1 ^A^ *	rs1894289	0.07	recessive	0.325	0.406	Total

False discovery rate (FDR) corrected P-values are shown for each association, and the mode under which the significant effect is observed is indicated. The percentage of affected and unaffected subjects carrying the minor allele is indicated.

A allelic test FDR also <0.20.

P-values:

* = 0.05–0.01 inclusive,

** = 0.0099–0.001 inclusive,

*** <0.001.

#### Haplotypes

The haplotype block analysis revealed that each gene was contained within a single haplotype block. Overall, association tests between haplotypes from each of the genes and the adult outcomes indicated that common haplotypes did not yield any more information than individual SNPs on the association between the studied genes and the phenotypes (not shown).

### Multivariate analyses

#### Gene-gene and gene-environment interactions

Given the extensive regulation of polyamine metabolism, we were next interested in determining if genetic factors found within separate genes (Table 2) may interact in conferring risk for psychiatric disorders. We found no significant interactions for either mood disorders or suicide attempts, as both the interaction terms between the two SNPs in mood disorders, and the three significant SNPs associated with suicide attempts, were not significant.

**Figure 1:**
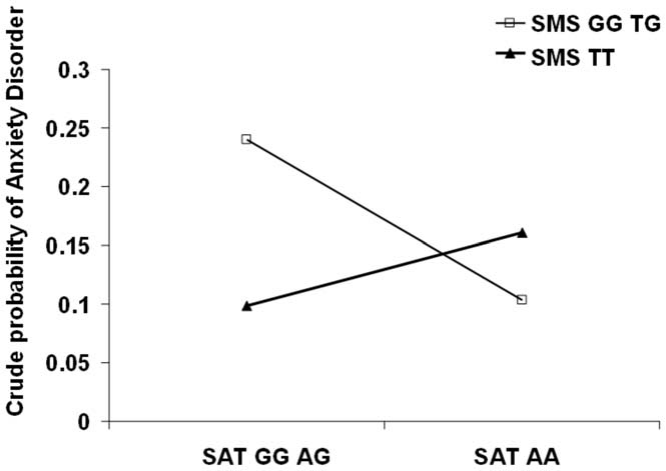
Significant gene-gene interaction between rs3764885 (SAT1) and rs6654100 (SMS) for anxiety disorders.

As there was a high correlation between two of the pairs of SNPs that were significantly associated with anxiety disorders (rs10521911 and rs6654100 in SMS: *r^2^* = 0.96; and rs6526342 and rs3764885 in SAT1: *r^2^* = 0.86), we randomly dropped one of the SNPs of each pair from the interaction analyses. We found a significant interaction in the additive model including the main effects and the interaction term of rs3764885 (SAT1) and rs6654100 (SMS) with respect to anxiety disorders (χ^2^_(1)_ = 6.25, P = 0.01). As shown in Figure 1, individuals with homo/heterozyosity for the G alleles in rs3764885 (SAT1) and in rs6654100 (SMS) had an elevated risk for anxiety disorders. No other interaction tests were significant for anxiety disorders.

**Figure 2:**
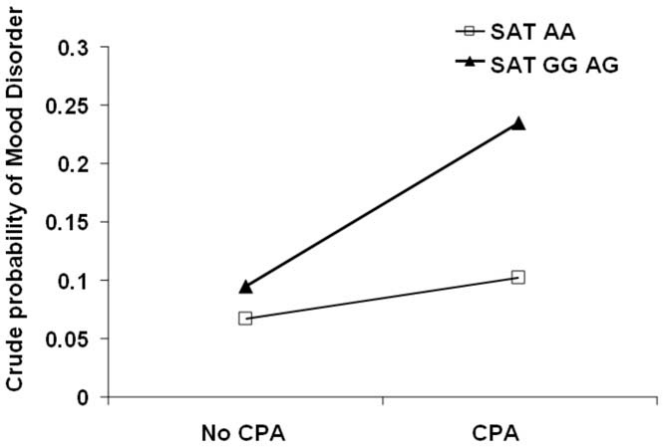
Significant gene-environment interaction between rs3764885 (SAT1) and childhood physical abuse (CPA) for mood disorders.

Next, we tested whether the significant SNPs for mood disorders found in the CPA subsample (Table 2) may yield significant gene-environment interactions in the overall sample. We found a significant interaction in the additive model including the main effects and the interaction term of rs3764885 (SAT1) and CPA as well as parental mood disorder (χ^2^_(1)_ = 5.51, P = 0.02). As depicted in Figure 2, individuals with homo/heterozyosity for the G allele in rs3764885 (SAT1) and who were CPA victims had elevated rates of mood disorders. This gene-environment interaction was not confounded by evocative gene-environment correlation as indicated by a nonsignificant correlation between the SNP and CPA (P = 0.75). In terms of passive gene-environment correlations, parental mood disorders were weakly associated with CPA (*r^2^* = 0.081, P = 0.005). No other gene-environment interactions were significant.

#### Main/mediating effects

Our final objective was to identify SNPs that are uniquely associated with our main outcomes, along with clinical variables which mediate these associations. The results of the multivariate analyses of the main effects are depicted in Table 3. Overall, the final logistic models explained approximately 18%, 14%, and 11% of the variance in mood disorders, suicide attempts and anxiety disorders, respectively. Using an equivalent of Cook's statistic for logistic regression, outlier diagnostics indicated no cases of extreme influence except, in the analysis of anxiety, the combination formed by the reference values of all variables. However, the latter represented the largest group of subjects. Multicollinearity was unlikely, as the highest correlation among our variables was *r^2^* = 10.8%. The final estimates obtained with the EM missing-value imputation method had narrower confidence intervals (CI) but were otherwise similar to unimputed estimates. Weighting for gender and family adversity did not change estimates of significance levels or odds ratios (*OR*) (not shown).

**Table 3:** Main and interactive effects for single nucleotide polymorphisms (SNP) and adult outcomes of mood disorders, suicide attempts, and anxiety disorders.

Phenotype	Gene	SNP	Effects, CI	Covariates	OR, 95%, CI
**Mood disorders**					
Model information D	SAT1	rs3764885	0.01, −0.02–0.05	Parental mood disorder	0.05, 0.01–0.10**
Omnibus test: χ^2^_(n = 1121, 5)_ = 87.14***				History of psychopathology A	0.07, 0.05–0.10***
Nagelkerke R^2^ = 0.16				CPA	0.00, −0.03–0.04
	SAT1	rs3764885 X CPA	0.10, 0.01–0.19*		
**Suicide attempts**					
Model information	OATL1	rs11795513	1.80, 1.20–2.71**	Parental suicide attempt	3.52, 1.84–6.72***
Omnibus test: χ^2^_(n = 1121, 3)_ = 80.68***				History of psychopathology B	2.74, 2.12–3.55***
Hosmer-Lemeshow test: χ^2^_(3)_ = 2.15, *p* = 0.54					
Nagelkerke R^2^ = 0.14					
**Anxiety disorders**					
Model information	SMS	rs5951676	1.96, 1.21–3.17**	Parental anxiety disorder	1.49, 0.91–2.45
Omnibus test: χ^2^_(n = 1121, 6)_ = 81.27***	SAT1	rs3764885	0.39, 0.29–0.77**	History of psychopathology C	2.03, 1.67–2.46***
Hosmer-Lemeshow test: χ^2^_(4)_ = 3.76, *p* = 0.44	SMS	rs6654100	0.34, 0.17–0.67**		
Nagelkerke R^2^ = 0.11	SAT1 X SMS	rs3764885 X rs6654100	5.39, 1.11–26.20*		

Effects and 95% confidence intervals (CI) are shown for each phenotype. Effects for mood disorders are represented by unstandardized regression coefficients, while effects for suicide attempts and anxiety disorders are represented by odds ratios.

A Anxiety, disruptive and substance abuse disorders, and suicide attempts.

B Anxiety, disruptive, mood and substance abuse disorders.

C Mood, disruptive and substance abuse disorders, and suicide attempts.

D Additive model.

P-values:

* = 0.05–0.01 inclusive,

** = 0.0099–0.001 inclusive,

*** <0.001.

#### Mood disorders

As mentioned above, rs3764885 and rs6526342 were highly associated, and were the only SNPs that were significant for the prediction of mood disorders in the overall sample. Table 3 depicts the final additive model that included standardized history of psychopathology in addition to the interaction model described above. We found no evidence for a mediating effect of CSA, although there was a relationship between CSA and mood disorders (*r* = 0.10, P = 0.0005), after controlling for the covariate effects.

#### Suicide attempts

One SNP (OATL1, rs11795513) made a statistically significant contribution to suicide attempts (*OR* = 1.80) independently of parental attempt (*OR* = 3.52) and Axis I diagnoses (*OR* = 2.74). We found evidence for evocative gene-environment correlation for this SNP and CSA (*r* = 0.10, P = 0.0008) as well as a link between CSA and suicide attempts (*r* = 0.17, P = 0.0001) after controlling for the covariate effects. After entering CSA in the regression model, the link between the OATL1 SNP and suicide attempt dropped (*OR* = 1.66, CI 1.09–2.51**). A Sobel test indicated significant (partial) mediation (Sobel statistic: *z* = 2.73, P = 0.0064; Goodman statistic *z* = 2.77, P = 0.0056). Parental suicide attempts were linked to CSA (*r* = 0.09, P = 0.0021) and CPA (*r* = 0.07, P = 0.0129). Although CPA was significantly linked to suicide attempts (*r* = 0.15, P<0.0001), it was nonsignificantly linked to the OATL1 SNP (*r* = −0.03, P = 0.3038), making further analyses unnecessary.

#### Anxiety disorders

In addition to the interaction effect of SAT1 (rs3764885) and SMS (rs6654100) described above, one SNP (SMS, rs5951676) made a statistically significant contribution to anxiety disorders (*OR* = 2.05), independently of the nonsignificant effect of parental anxiety disorders (*OR* = 1.05) and the significant effect of history of psychopathology (*OR* = 1.14). Neither CSA nor CPA demonstrated mediating effects for anxiety disorders.

#### Candidate endophenotypes

Externalizing (disruptiveness) symptoms were significantly linked to mood disorders (*r* = 0.16, P<0.0001), anxiety disorders (*r* = 0.09, P = 0.0024), and suicide attempts (*r* = 0.21, P<0.0001). Disruptiveness was also linked to the OATL1 SNP rs11795513 (*r* = 0.09, P = 0.0042) that was linked to suicide attempts (see Table 3). After entering disruptiveness in the regression model predicting suicide attempts, the relationship between the OATL1 SNP and the outcome dropped (*OR* = 1.71, CI 1.13–2.59**). A Sobel test indicated significant (partial) mediation (Sobel statistic: *z* = 2.25, P = 0.0244; Goodman statistic *z* = 2.30, P = 0.0215). Disruptiveness did not demonstrate mediating effects with respect to mood disorders or anxiety disorders, although it was significantly linked to rs3764885 (SAT1) (*r* = −0.09, P = 0.0009) which predicted anxiety disorders and mood disorders.

Neither of the internalizing endophenotypes demonstrated mediating effects with respect to any of the three outcomes.

## Discussion

In this study, we examined the relationships between genetic variants in polyamine genes and three main outcomes: suicide attempts, mood disorders, and anxiety disorders. In addition, we assessed the gene-gene and gene-environment interactions as well as investigated the involvement of several potential endophenotypes as mediators for our genetic effects. Overall, not only did each of the four genes examined demonstrate significant associations with at least one of our main outcomes, but each of these outcomes was in turn associated with polyaminergic variants.

Among the genes examined, SAT1 displayed the greatest range of effects, with polymorphisms demonstrating significant associations with each of the three main outcomes. The SAT1 gene encodes the rate-limiting enzyme in polyamine catabolism, and has provided the most compelling evidence for the involvement of dysregulated polyamine catabolism in psychiatric conditions, particularly in completed suicide where it displays widespread decreases in expression across the brain [21], [22], [24], [25]. In the present study, three SAT1 SNPs, rs6526342, rs3764885, and rs1894289, demonstrated significant associations with the main outcomes; interestingly however, our results indicate disease-specificity with respect to the risk alleles at these polymorphisms. Indeed, while rs6526342 and rs3764885 were significantly associated with both mood and anxiety disorders, the major alleles at these SNPs represented the risk alleles in anxiety disorders, whereas the minor alleles conferred susceptibility towards mood disorders. Both these SNPs, as well as two other polymorphisms, rs6151267 and rs1960264, are found on the same haplotype block [31], and greater proportions of the major haplotype at this locus have been associated with both completed suicide and anxiety disorders [21], [28], [29]. Interestingly, the association of rs6151267 with completed suicide was only found when comparing individuals with depressive disorders, indicating that the risk conferred by this polymorphism is specific to suicide performed in the context of depression. The results of the present study may thus help explain this effect, as it could be that the ability of this haplotype to confer susceptibility to suicide may have been more apparent in a sample in which the opposite allele is found at higher proportions. At the molecular level, we recently characterized the role of this haplotype in influencing the expression of SAT1, and found that the more common haplotype was associated with lower gene expression in vitro and in the brain [31]. Accordingly, it could be speculated that the relationships between these SNPs and mood and anxiety disorders are mediated by the effects of this haplotype on gene expression. Another SAT1 SNP, rs1894289, also possessed disease-specific risk alleles in terms of its associations with attempted suicide and mood disorders. Given that this SNP is located approximately 3.5 kb downstream of SAT1, it is unlikely that it possesses a functional role, and rather is tagging the functional variant. Compared to the other SAT1 SNPs examined, the linkage disequilibrium between this SNP and others on the haplotype block is much lower: it is therefore quite possible that this SNP is tagging an adjacent haplotype block, and that the associations of this SNP involve distinct molecular mechanisms.

In this study, SMS, which is involved in spermine biosynthesis, demonstrated strong associations with anxiety disorders through both main genetic effects as well as through a gene-gene interaction with SAT1. Although our previous results had indicated that this gene demonstrates altered expression in the hippocampus of suicide completers [22], this represents the first study indicating that it may also be involved in anxiety. We recently characterized genetic and epigenetic factors in the promoter region of SMS and found no indication that promoter variants, DNA methylation, or chromatin modifications played a role in determining the expression of this gene [59]. However, as all of our significant associations in the present study map to introns or regions downstream from SMS, these results may indicate that genetic variants outside of the promoter region could influence the expression of this gene. Alternatively, the functional variants responsible for the associations with anxiety may influence later gene regulatory steps, such as mRNA processing or enzymatic activity. Indeed, several rare mutations in SMS, resulting in altered splicing or enzymatic activity, are responsible for Snyder-Robinson syndrome, a form of X-linked mental retardation which manifests with both intellectual and physical symptoms including alterations in brain morphology [60]–[63]. Certainly additional studies will be required to identify the variants which are responsible for our associations as well as the mechanisms by which they exert their effects. In addition to the main genetic effects, we identified a gene-gene interaction between SMS and SAT1 in conferring risk for anxiety disorders. Interestingly, SMS and SAT1 are located in close proximity on the X chromosome, and are at a chromosomal region which was recently found to be associated with suicide [64]. This could indicate the involvement of shared regulatory mechanisms, which may be involved in the interactive effects between SNPs in these two genes.

Unlike the other genes included in this study, the significant findings regarding SMOX and mood disorders were only apparent in the subset of individuals who had experienced physical abuse in childhood. Within this subgroup, three SNPs, two within the third intron and one located 3 kb downstream, demonstrated significant associations with mood disorders. Along with SMS, we recently examined genetic and epigenetic elements in the SMOX promoter, and found little indication that expression of this gene was influenced through these mechanisms [59]. Aside from our previous findings regarding altered expression of this gene in suicide completers [25], little is known concerning the involvement of this gene in psychiatric conditions. As such, the mechanisms by which these SNPs may act to confer susceptibility to mood disorders is unknown.

OATL1 yielded the strongest univariate associations in this study, with two SNPs being significantly associated with suicide attempts. Compared to the other genes in this study, the relationship of OATL1 to polyamine metabolism is more distant, and its physiological function or role in polyamine metabolism has not been well characterized. This gene was selected for this study due to its potential involvement in suicide, as it displays altered expression in the hippocampus of suicide completers [22]. While the current study appears to support its involvement in suicidal behaviors, the mechanism by which it exerts its pathological effects remains unclear. Indeed, the two SNPs associated with attempted suicide map outside of the gene, with rs11795513 located approximately 2 kb upstream and rs2249583 approximately 13 kb downstream. These SNPs are part of a larger haplotype block on the X chromosome which encompasses several genes [38], [65], and as such, it is also possible that these associations may be due to genes located nearby rather than OATL1.

In addition to main genetic effects and gene-gene interactions, this study examined the role of gene-environment interactions in conferring risk towards each of the three main outcomes, as well as controlled for possible confounding effects of gene-environment correlations. Our results demonstrated that exposure to either physical or sexual abuse was associated with elevated risk for suicide attempts, mood disorders, and anxiety disorders, however our results indicated that sexual abuse did not moderate the genetic associations of the polyamine genes with these outcomes. Our results identified a significant moderating effect of physical abuse on the association between the SAT1 SNP rs3764885 and mood disorders. Particularly interesting is that while higher levels of the minor allele were associated with mood disorders in the univariate analyses, the effects of physical abuse were to increase the risk for mood disorders in individuals carrying the major allele. Finally, we identified several evocative gene-environment correlations with regards to CSA and its involvement in mood disorders and suicide attempts, however the mechanisms involved in these effects are not clear.

Along with analyses investigating the relationship between genetics and the environment, a growing trend in psychiatric research has been to investigate endophenotypes as a means to disentangle the relationships between genetic variables and psychiatric disorders. Whereas our previous findings demonstrated that externalizing and internalizing trajectories measured in childhood were not suitable endophenotypes for mood disorders or suicidal behaviors [36], [37], the present study demonstrates that measurement of these symptoms at an older age may be more appropriate for describing suicidal behaviors. The ability of externalizing behaviors to act as an endophenotype to explain the relationship between our genetic findings and suicide attempts agrees well with the strong association between impulsive aggression and suicidal behaviors [15]. In addition, these results agree with our previous findings that trajectories based upon disruptiveness demonstrate a stronger relationship with suicide attempts than those derived from measures of anxiousness [37]. We found no evidence that our externalizing or internalizing endophenotypes mediate the relationships between our genetic findings and mood or anxiety disorders. This may reflect a lack of statistical power in our sample to detect these effects, or alternatively, these relationships may be mediated by other personality measures.

There are several potential limitations to this study. Although we removed perfectly correlated SNPs from our analyses in order to reduce statistical corrections, it is clear that many of the remaining SNPs were highly correlated. As such, we likely overcorrected for multiple testing in using the Benjamini–Hochsberg procedure and may have missed additional weaker associations. Moreover, as with all studies of this nature, the presence of linkage disequilibrium prevents us from conclusively identifying the functional variants nor the mechanisms by which they exert their effects. Additionally, our statistical power to characterize genetic effects in the smaller CPA and CSA subsamples was limited and may have prevented us from identifying gene-environment interactions. Also, as CSA and CPA were assessed by self-report, recall bias may have affected these measurements. Finally, as we did not correct our interaction analyses for multiple testing, these findings should thus be interpreted with caution, as they are in need of replication.

In conclusion, this study identified a number of genetic and environmental variables associated with attempted suicide, mood disorders, and anxiety disorders. While the precise biological mechanisms by which these genetic variants confer risk to these disorders remains to be determined, these studies have greatly extended our knowledge regarding the involvement of dysregulated polyamine metabolism in the etiology and pathology of psychiatric conditions.

## Supporting Information

Figure S1 **Linkage disequilibrium (r^2^) between polymorphisms within each gene.**
(DOC)

Table S1 **Single nucleotide polymorphisms genotyped in each gene.** Major and minor alleles are shown for each variant.
(DOC)

Table S2 **Power analyses for association testing with mood disorders in the total sample and the subgroup of individuals exposed to childhood physical abuse (CPA), under the assumption of an interaction between genotype and CPA.** Tests were computed under the dominant genetic model by combining carriers (homozygotes and heterozygotes) of the risk allele. Power calculations were assessed for α levels corresponding to false discovery rates (FDR) of 0.2.
(DOC)

Table S3 **Power analyses for regression analyses of the interaction between a genotype and childhood physical abuse (CPA) on mood disorders.** Tests were computed under the dominant genetic model by combining carriers (homozygotes and heterozygotes) of the risk allele. Power calculations were assessed for α = 0.05.
(DOC)
