# Supplementary figures and images for: Association of Polyaminergic Loci With Anxiety, Mood Disorders, and Attempted Suicide
Source: PLoS One. 2010 Nov 30;5(11):e15146. doi: 10.1371/journal.pone.0015146 (PMC2994870; doi:10.1371/journal.pone.0015146)

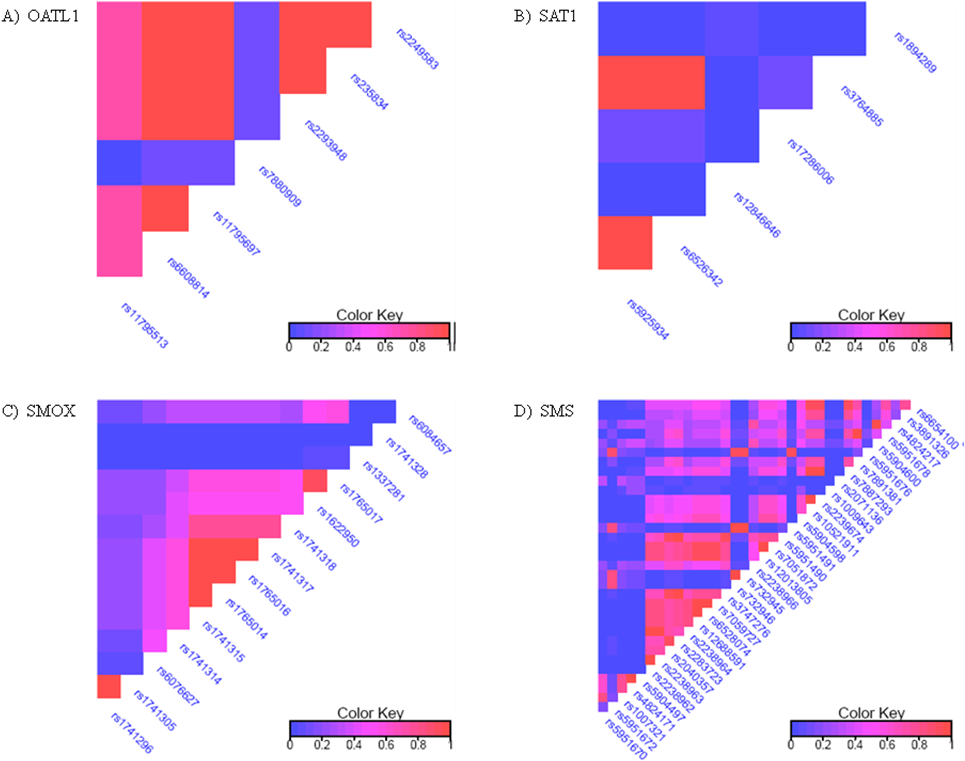


Supplementary Figure S1: Linkage disequilibrium (r2) between polymorphisms within each gene.

Supplement: Figure S1 — Linkage disequilibrium (r2) between polymorphisms within each gene. (DOC) [file pone.0015146.s001.doc]
