# Supplementary material for: Association of Polyaminergic Loci With Anxiety, Mood Disorders, and Attempted Suicide
Source: PLoS One. 2010 Nov 30;5(11):e15146. doi: 10.1371/journal.pone.0015146 (PMC2994870; doi:10.1371/journal.pone.0015146)
Supplement: Table S1 — Single nucleotide polymorphisms genotyped in each gene. Major and minor alleles are shown for each variant. (DOC) [file pone.0015146.s002.doc]

Supplementary Table S1: Single nucleotide polymorphisms genotyped in each gene. Major and minor alleles are shown for each variant.

| **OATL1** | **SAT1** | **SMOX** | **SMS** |
| --- | --- | --- | --- |
| rs11795513 (C/T) | rs5925934 (T/C) ^B^ | rs1741296 (A/G) | rs5951670 (G/A) |
| rs6608814 (T/C) ^B^ | rs6526342 (C/A) | rs1741305 (C/T) | rs2238958 (G/A) ^A^ |
| rs11795697 (G/A) ^B^ | rs17286006 (C/T) | rs6076627 (A/T) | rs5951672 (G/A) |
| rs7880909 (G/A) | rs3764885 (G/A) | rs1741314 (C/T) | rs1007321 (C/G) |
| rs2293948 (A/G) ^B^ | rs1894289 (T/G) | rs1741315 (G/A) | rs4824171 (G/A) |
| rs235834 (T/A) ^B^ | rs12846646 (G/A) | rs1765014 (A/G) ^B^ | rs5904497 (T/G) ^B^ |
| rs2249583 (G/C) |  | rs1765016 (G/T) ^B^ | rs2238962 (G/A) |
|  |  | rs1741317 (G/A) ^B^ | rs2238963 (G/A) ^B^ |
|  |  | rs1741318 (G/A) | rs2040357 (G/A) |
|  |  | rs1535225 (G/A) ^A^ | rs2283723 (A/G) |
|  |  | rs1622950 (A/G) | rs2238964 (C/T) |
|  |  | rs1765017 (T/G) | rs12688591 (A/C) |
|  |  | rs1337281 (G/T) | rs6528074 (G/A) ^B^ |
|  |  | rs1741328 (C/T) | rs7059727 (C/T) ^B^ |
|  |  | rs6084657 (T/C) | rs3747276 (C/T) |
|  |  |  | rs732946 (G/A) |
|  |  |  | rs732945 (C/G) ^B^ |
|  |  |  | rs2238966 (C/T) |
|  |  |  | rs12013805 (G/T) |
|  |  |  | rs7051872 (C/G) ^B^ |
|  |  |  | rs5951490 (A/C) |
|  |  |  | rs5951491 (G/A) ^B^ |
|  |  |  | rs5904598 (A/T) |
|  |  |  | rs10521911 (C/T) |
|  |  |  | rs2239674 (A/G) ^B^ |
|  |  |  | rs1009643 (T/C) |
|  |  |  | rs2071136 (T/C) |
|  |  |  | rs7887293 (G/T) ^B^ |
|  |  |  | rs7891381 (A/G) ^B^ |
|  |  |  | rs5951676 (T/C) |
|  |  |  | rs5904600 (G/A) |
|  |  |  | rs5951678 (G/A) |
|  |  |  | rs4824217 (T/G) |
|  |  |  | rs3891326 (G/A) |
|  |  |  | rs6654100 (G/T) |

^A^ Excluded due to call rate below 90%

^B^ Excluded due to perfect (r=1.0) correlation with at least one other SNP in this list.
