# Supplementary material for: Association of Polyaminergic Loci With Anxiety, Mood Disorders, and Attempted Suicide
Source: PLoS One. 2010 Nov 30;5(11):e15146. doi: 10.1371/journal.pone.0015146 (PMC2994870; doi:10.1371/journal.pone.0015146)
Supplement: Table S2 — Power analyses for association testing with mood disorders in the total sample and the subgroup of individuals exposed to childhood physical abuse (CPA), under the assumption of an interaction between genotype and CPA. Tests were computed under the dominant genetic model by combining carriers (homozygotes and heterozygotes) of the risk allele. Power calculations were assessed for α levels corresponding to false discovery rates (FDR) of 0.2. (DOC) [file pone.0015146.s003.doc]

**Supplementary Table S2: Power analyses for association testing with mood disorders in the total sample and the subgroup of individuals exposed to childhood physical abuse (CPA), under the assumption of an interaction between genotype and CPA.**

| **Genotype attributable risk** | **Frequency of risk allele (%)** | **Relative risk ^*^** | **Power (%)** | |
| --- | --- | --- | --- | --- |
|  |  |  | **CPA (α=0.05)** | **Total (α=0.005)** |
| 0.10 | 0.05 | 3.84 | 99 | 62 |
|  | 0.10 | 2.46 | 86 | 27 |
|  | 0.30 | 1.54 | 42 | 5 |
|  | 0.50 | 1.37 | 23 | 1 |
| 0.20 | 0.10 | 5.04 | 100 | 97 |
|  | 0.30 | 2.50 | 91 | 57 |
|  | 0.50 | 2.02 | 48 | 30 |

Tests were computed under the dominant genetic model by combining carriers (homozygotes and heterozygotes) of the risk allele. Power calculations were assessed for α levels corresponding to false discovery rates (FDR) of 0.2.

^*^ Relative risk of mood disorders between carriers and non-carriers of the risk genotype in the CPA subgroup. The relative risk in the non-CPA subgroup was equal to 1.
