# Supplementary material for: Association of Polyaminergic Loci With Anxiety, Mood Disorders, and Attempted Suicide
Source: PLoS One. 2010 Nov 30;5(11):e15146. doi: 10.1371/journal.pone.0015146 (PMC2994870; doi:10.1371/journal.pone.0015146)
Supplement: Table S3 — Power analyses for regression analyses of the interaction between a genotype and childhood physical abuse (CPA) on mood disorders. Tests were computed under the dominant genetic model by combining carriers (homozygotes and heterozygotes) of the risk allele. Power calculations were assessed for α = 0.05. (DOC) [file pone.0015146.s004.doc]

**Supplementary Table S3: Power analyses for regression analyses of the interaction between a genotype and childhood physical abuse (CPA) on mood disorders.**

| **Genotype attributable risk** | **Frequency of risk allele (%)** | **Relative risk ^*^** | **Power (%)** | |
| --- | --- | --- | --- | --- |
|  |  |  | **Additive** | **Logistic** |
| 0.10 | 0.05 | 3.84 | 95 | 100 |
|  | 0.10 | 2.46 | 76 | 100 |
|  | 0.30 | 1.54 | 32 | 83 |
|  | 0.50 | 1.37 | 15 | 32 |
| 0.20 | 0.10 | 5.04 | 100 | 100 |
|  | 0.30 | 2.50 | 86 | 100 |
|  | 0.50 | 2.02 | 51 | 80 |

Tests were computed under the dominant genetic model by combining carriers (homozygotes and heterozygotes) of the risk allele. Power calculations were assessed for α = 0.05.

^*^ Relative risk of mood disorders between carriers and non-carriers of the risk genotype in the CPA subgroup. The relative risk in the non-CPA subgroup was equal to 1.
